# Emotion regulation shows an age- and sex-specific moderating effect on the relationship between chronic stress and cognitive performance

**DOI:** 10.1038/s41598-024-52756-3

**Published:** 2024-02-06

**Authors:** Jan S. Novotný, Luka Srt, Gorazd B. Stokin

**Affiliations:** 1 Institute of Molecular and Translational Medicine, Faculty of Medicine and Dentistry, Palacký University Olomouc, Hněvotínská 1333/5, 779 00 Olomouc, Czech Republic; 2 Faculty of Medicine, University of Maribor, Maribor, Slovenia; 3 Department of Neurology, Gloucestershire Hospitals NHS Foundation Trust, Gloucester, UK; 4 Translational Neuroscience and Aging Program, Center for Translational Medicine, International Clinical Research Centre, St. Anne’s University Hospital, Brno, Czech Republic

**Keywords:** Cognitive ageing, Cognitive neuroscience, Emotion, Stress and resilience, Human behaviour

## Abstract

Despite the extensive knowledge about the effects of chronic stress on cognition, the underlying mechanisms remain unclear. We conducted a cross-sectional moderation analysis on a population-based sample of 596 adults to examine the age- and sex-specific role of emotion regulation (ER) in the relationship between chronic stress and cognitive performance using validated self-report questionnaires. While women showed no direct or moderated relationship between stress and cognition, men displayed a distinct age-related pattern where stress was negatively associated with poorer cognitive performance at older ages, and the onset of this relationship was detected earlier in men with ER problems. These results showed that suppression of emotions and lack of executive control of ER amplify the negative consequences of chronic stress and suggest that there are sex-specific differences in the decline of ability to cope with long-term exposure to stressors.

## Introduction

In today's rapidly changing world, affected in recent years by several major crises such as the Covid-19 pandemic, the war in Ukraine and the related energy and cost of living crises, individuals are invariably exposed to various stressors. This commonly leads to increased experience of stress^1–3^. Defined as a state of threatened homeostasis following exposure to extrinsic or intrinsic adverse forces^4^, this stress can negatively affect daily functioning. In contrast to short-term acute stress, which can have both positive and negative effects, chronic stress acting over a prolonged period has been found to exert primarily negative effects in several areas including mental health and cognitive performance^5–9^.

The major pathways activated by stress include the hypothalamic–pituitary–adrenal (HPA) axis^10^ and the autonomic nervous system (ANS)^11^. The sympathetic nervous system (SNS) first activates the fight or flight response to deal with stressors including the production of catecholamines^12^. Prolonged SNS activation without parasympathetic nervous system counteraction, however, then leads to the activation of the immune system including the production of pro-inflammatory cytokines^13^. Activation of the HPA axis, the neuroendocrine circuit, in which limbic and hypothalamic brain structures coordinate emotional, cognitive, neuroendocrine and autonomic inputs and responses on the other hand leads to increased production of glucocorticoids^14^ with further SNS-driven involvement of catecholamines. Long-term activation of these pathways can then lead to a reduction in the volume and plasticity of brain regions such as the prefrontal cortex, hippocampus, amygdala and basal ganglia^14,15^.

These stress-related neurophysiological changes subsequently translate into behavioral, cognitive and emotional responses to stress and its consequences. Previous studies have demonstrated that chronic stress is linked to negative behavioral and emotional responses such as anxiety^5^, depression^6,16^, or burnout^7,17^, as well as having an effect on cognitive domains such as learning and memory^8,9,18–20^, attention^21^, and executive function^9^. These diverse effects of chronic stress stem from the fact that all these processes share the same neural pathways and brain regions including the amygdala, hippocampus, and prefrontal cortex (PFC)^14,22,23^.

Mounting evidence suggests that the relationship between stress and cognition is further moderated by a range of other factors. One of them is the emotion regulation (ER), which refers to the process of controlling and modifying emotional responses to environmental stimuli to accomplish goals^24^. This process can occur at both unconscious and conscious levels^25^ and generally involves several steps: the ability to recognize the emotional significance of a stimulus, assess the need for regulation, select an appropriate strategy and apply it^26^. Several different dimensions/strategies are available for emotion regulation. Augustine and Hemenover^27^ described two basic types of strategies: behavioral and cognitive. Similarly, Aldo et al.^28^ reported six distinct strategies (acceptance, avoidance, problem solving, reappraisal, rumination, and suppression) that can be assigned to the above two basic types. Finally, Gratz and Roemer^29^ inversely outlined six subtypes of possible difficulties with ER that form the basis for this study (nonacceptance of emotional responses, difficulty engaging in goal-directed behavior, impulse control difficulties, lack of emotional awareness, limited access to emotion regulation strategies, and lack of emotional clarity).

The rationale for the close link between ER, chronic stress and cognition derives from several facts. Neurophysiologically, they share neural pathways and brain regions^30^, the effect of HPA axis-related cortisol that may reduce PFC activity and impair hippocampal-dependent memory^31^, as well as positive effects of cortisol on ER during stress^32,33^. From a psychological standpoint, ER is tightly linked to some coping strategies. Coping strategies refers to constantly changing cognitive and behavioral efforts to manage specific demands that are taxing or exceeding the individual’s resources^34^. These strategies can be broadly divided into three categories: problem-focused (direct problem-solving efforts), emotion-focused (management of stress-related emotions) and appraisal-focused (cognitive assessment and logical analysis)^35^. Although coping and ER are perceived as distinct constructs^30,36^, they are also closely intertwined and ER can intervene directly or indirectly in all three areas of coping. The most explicit link is with emotion-focused coping as it provides tools directly designed to manage stress-related emotions^37–39^. Furthermore, reappraisal (as one of the ER strategies) may, for example, help change the emotional perception of a stressful situation into a positive, less burdensome direction^40^. Finally, reducing stress-induced emotional and cognitive load then indirectly frees mental resources for direct problem solving.

However, so far, the main research interests have focused on the role of cognition in the regulation of stress-related emotional responses. The studies emphasized the role of cognition in emotional experience and ER^41,42^ and showed that stress affects the use of cognitive processes in controlling emotional reactions^43^ and leads to differential activation of the left frontal lobe during cognitive regulation of emotion^44^. Conversely, evidence on whether and how ER affects the relationship between stress and cognitive performance is sparse. For example Kalia and Knauft observed that ER strategies moderate the negative effect of adverse childhood experiences on cognitive flexibility in adulthood^45^. Overall, however, the role of ER in this relationship is not yet fully understood.

In addition, the effect of other aspects such as sex and age in this relationship may also play a significant role and have not yet been fully explored. For example, previous studies showed that men prefer problem-focused coping, which is more cognitively dependent, while women tend to favor emotion-focused coping^46–51^. Similarly, sex-related differences in ER^52,53^ and in cognitive performance^54–56^ have been demonstrated in multiple studies. In the context of age-related differences, previous studies have yielded inconsistent findings regarding the direction of changes in the ER, but have confirmed the presence of these changes over the lifespan^57–60^. The gradual decline of some cognitive resources with age (which may influence the effectiveness of ER and coping strategies) and cognitive abilities in general has also been extensively described^61–67^.

To fill this gap, we examine the age- and sex-specific role of ER in the relationship between chronic stress and cognitive performance in a probabilistic population-based sample. To achieve this goal, we conducted an exploratory cross-sectional moderation analysis of age-stratified data collected using a battery of validated self-report questionnaires measuring chronic stress (Perceived Stress Scale), Emotion Regulation (Difficulties with Emotion Regulation Scale), and cognitive performance (Montreal Cognitive Assessment).

## Results

### Sample characteristics

The sample included a total of 569 adults with a mean age ± SD of 55.76 ± 10.74 years, of whom 290 (51%) were women. The mean years of education ± SD was 15.7 ± 3.3 years. The main age-stratified characteristics of the participants are shown in Table 1.

**Table 1:** Age-stratified socio-demographic characteristics of participants.

	Age group		
	33–49	50–64	65 +
N	193	220	156
Age ± SD	43.2 ± 4.2	57.5 ± 4.2	68.8 ± 2.3
Females, N(%)	99 (51.3%)	109 (49.5%)	82 (52.6%)
Avg. education, years ± SD	16.1 ± 3.6	15.3 ± 3	15.6 ± 3.3
Employment, N (%)			
Yes, full-time	167 (86.5)	171 (77.7)	19 (12.2)
Yes, part-time	19 (9.8)	19 (8.6)	31 (19.9)
No	7 (3.6)	30 (13.6)	106 (67.9)
Household monthly income (in CZK), N (%)			
< 15 k	2 (1)	9 (4.1)	9 (5.8)
15–30 k	24 (12.4)	37 (16.8)	55 (35.3)
30–45 k	36 (18.7)	69 (31.4)	56 (35.9)
45–60 k	51 (26.4)	47 (21.4)	21 (13.5)
> 60 k	78 (40.4)	52 (23.6)	12 (7.7)
No answer	2 (1)	6 (2.7)	3 (1.9)

### Preliminary verification of the relationship between stress and cognition

**Figure 1:**
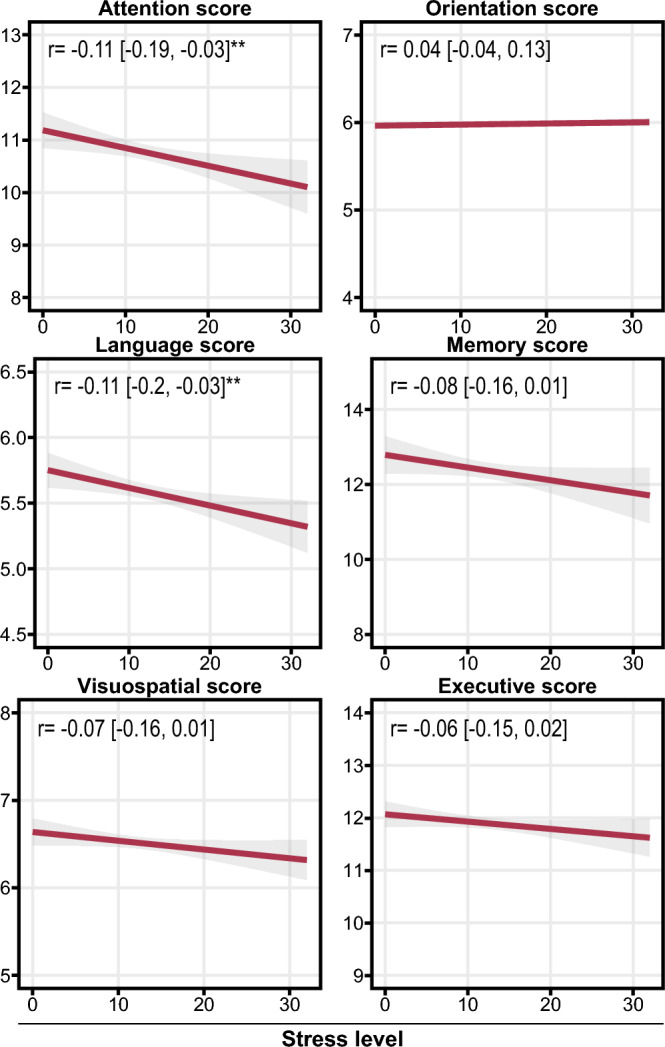
Correlation between perceived stress and cognitive performance. Regression lines showing Pearson correlations between stress level and MoCA total score and individual Cognitive Domain Index Scores. The r values with 95% confidence intervals are embedded. Asterisks indicate the significance (**P < 0.01). N = 569.

To test whether our results are consistent with previous reports, we first investigated whether our population-based sample also exhibits an association between stress and cognitive performance (Fig. 1, Supplementary Table T1). We observed significant correlation between the Perceived Stress Scale (PSS) and the Montreal Cognitive Assessment (MoCA) Attention (r [95% CI] = − 0.11 [− 0.19, − 0.03]) and Language (r [95% CI] = − 0.11 [− 0.2, − 0.03]) Cognitive Domain Index Scores (CDIS) (r [95% CI] = − 0.12 [− 0.2, − 0.03]). All of these correlations were negative suggesting that increased stress is linked to impairment in cognitive performance.

### Age- and sex-related distribution of ER and cognition

**Figure 2:**
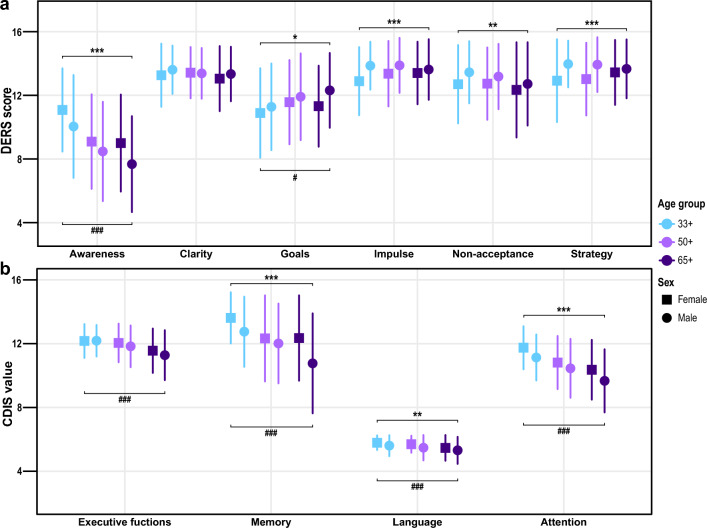
Age and sex-dependent differences in emotion regulation and MoCA Cognitive Domain Index Scores. The point plot shows the mean values of the (**a**). individual domains of emotion regulation difficulties (DERS) and (**b**). MoCA CDIS stratified by age and sex. Higher DERS scores indicate a greater presence of emotion regulation difficulties. Higher MoCA CDIS scores indicate better cognitive performance. Error bars indicate the standard deviation. Significant differences between groups by age and sex were analyzed using two-way ANOVA. Upper asterisks show significant simple main effects between sexes (*P < 0.05, **P < 0.01, ***P < 0.001), and lower hashtags indicate significant simple main effects between age groups (^#^*P* < 0.05, ^###^*P* < 0.001).

We next examined the distribution of ER difficulties scores in respect to aging and sex (Fig. 2A, Supplementary Tables T2 and T3). Apart from Awareness (η^2^_P-value_ = 0.09_<0.001_, ibid.) and Goals (η^2^ = 0.01_0.015_), there were no age-related differences in ER difficulties. In contrast, we found significant sex differences in all domains except for Clarity. Men exhibited greater ER difficulties in Goals (η^2^ = 0.01_0.016_), Impulse (η^2^ = 0.02_<0.001_), Non-acceptance (η^2^ = 0.01_0.007_) and Strategy (η^2^ = 0.03_<0.001_), while women demonstrated greater difficulties in Awareness (η^2^ = 0.02_0.007_). Similarly, we analyzed the age- and sex-specific distribution of MoCA CDIS that represent cognitive resources for ER (Fig. 2B, Supplementary Tables T4 and T5). We observed a significant age-related decline in all CDIS (Exec. functions η^2^ = 0.05_<0.001_, Memory η^2^ = 0.06_<0.001_, Language η^2^ = 0.03_<0.001_, Attention η^2^ = 0.10_<0.001_) and significantly lower scores for men in Memory (η^2^ = 0.03_<0.001_), Language (η^2^ = 0.02_<0.001_) and Attention (η^2^ = 0.02_<0.001_) compared to women.

### Moderation analysis of the role of ER in the relationship between stress and cognition

**Figure 3:**
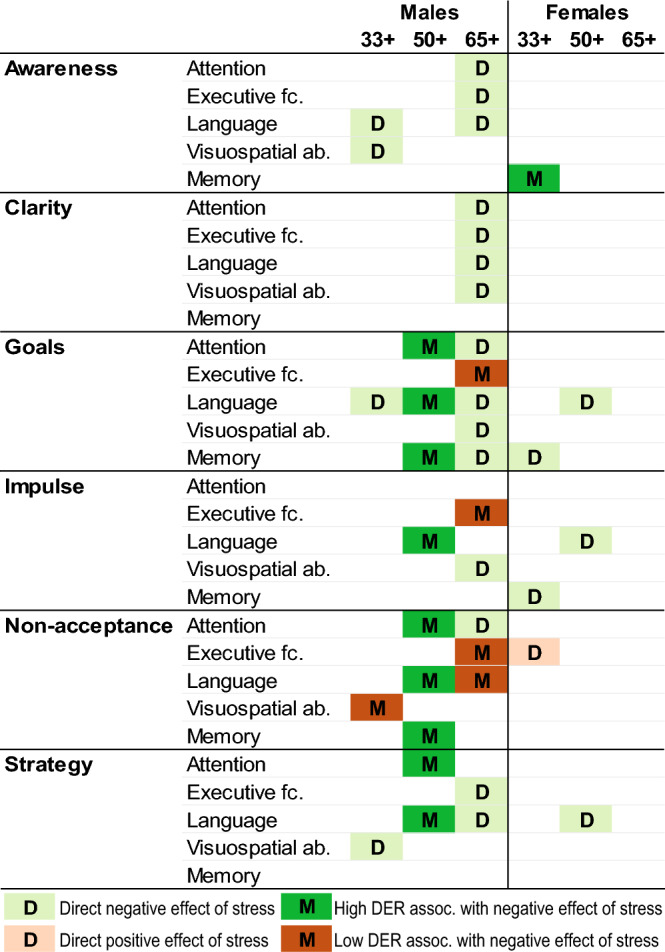
The moderating role of emotion regulation in the relationship between stress and cognitive performance. Summary of the identified direct effects of stress on cognitive performance and the moderating effects of emotion regulation difficulties on this effect by sex and age. Colored squares in the matrix identify combinations of emotional regulation difficulty domains and cognitive domains in which a direct negative/positive effect of stress on a given cognitive domain or a moderating effect of difficulties with emotional regulation (DER) on the relationship between stress and cognitive performance was present. The moderating effect of the presence of emotion regulation difficulties either increased or decreased the negative impact of stress on a given cognitive domain. All observed moderation effects represented full moderation.

Finally, we tested whether ER moderates the impact of stress on cognition (Fig. 3, Supplementary Table T6). In women, we found only one moderating and few direct effects associated mainly with memory (33 + years group) and language (50 + years group). All but one of the direct effects were negative, suggesting that cognitive performance deteriorates with increasing stress (with std. B ranging from -0.019 to -0.054 for negative effects and std. B = 0.034 for the positive effect of stress on executive function in the 33 + years group). ER difficulties (namely awareness) moderated only the effect of stress on memory in the 33 + years group (std. B = − 0.016).

In contrast, an age-dependent role of ER on the interaction between stress and cognition was observed in men (Fig. 3, Supplementary Table T6). In the 33 + years old age group, we observed almost no direct nor moderating effect of stress on cognitive performance. With advancing age (50 + years old age group), the relationship between stress and cognitive performance depended on the presence of ER difficulties. Finally, in the oldest 65 + years old age group, the cognitive performance was mostly directly linked to stress level with emotional regulation maintaining a moderating role in only a few instances. All observed moderation effects represented full moderation with no significant direct effect of stress.

**Figure 4:**
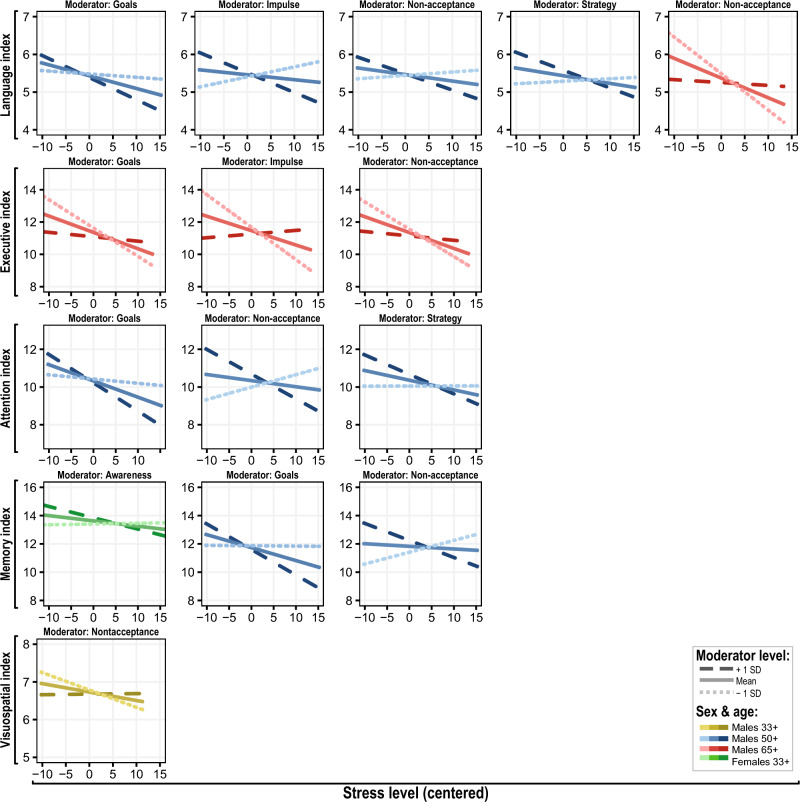
Simple slopes of the significant moderating roles of emotion regulation difficulties in the relationship between stress and cognitive domains. Simple slopes of the identified significant moderating effects of emotional regulation difficulties on the impact of stress on cognitive scores in individual cognitive domains. The individual regression lines simulate the effect of stress on cognitive domain score when the given emotion regulation difficulty score is anchored to the mean, and ± 1 SD from that mean. Values of emotional regulation difficulties and stress levels are centered.

More specifically, in the 50 + years old age group, the ER difficulties in Goals, Non-acceptance, Strategy, and Impulse moderated the extent to which stress was negatively associated with cognitive performance in Attention (with interaction std. B ranging from − 0.023 to − 0.049), Language (std. B: − 0.009 to − 0.026) and Memory (std. B: − -0.033 to − 0.051). The analysis of simple slopes revealed that higher presence of these ER difficulties (+ 1SD above mean) was associated with significant negative relationship between stress and individual CDIS (Fig. 4, Supplementary Table T7). In the 65 + years old age group, the stress was directly negatively associated with Attention (std. B of main effect in individual models ranging from − 0.091 to − 0.122), Executive functions (std. B: − 0.08 to − 0.084), Language (std. B: − 0.04 to − 0.046) and Visuospatial abilities (std. B: − 0.039 to − 0.043). For the documented moderating effect of ER difficulties, we observed that Goals, Impulse, and Non-acceptance moderated the association between stress and Executive functions (std. B = 0.031, 0.059, and 0.027), and Non-acceptance further moderated the association between stress and Language (std. B = 0.017). In stark contrast to the 50 + years group, simple slopes analysis (Fig. 4, Supplementary Table T7) showed that in the 65 + years group, stress was significantly negatively correlated with individual cognitive scores in participants with lower (− 1SD below mean) and average ER difficulties, but not for those with greater difficulties.

## Discussion

As our society continues to face diverse sources of stress (currently amplified by the global crises of recent years), understanding the mechanisms how it affects cognitive functioning is of critical importance. Despite a growing body of research on stress and cognition, the moderating role of ER received only little attention. Similarly, little is known about the role of age and sex in this moderating role of ER. Our study, one of the first on this topic, revealed striking age- and sex-related differences in the pattern of both the direct and moderating effects. Women showed no effects of stress nor ER difficulties on cognition. In contrast, men displayed a distinct age-related pattern where difficulties with emotion regulation accelerated the onset of a negative relationship between chronic stress and cognitive performance.

In detail, we observed that a significant relationship between stress and cognition emerged first in men with problems in several areas of ER. Specifically, these included non-acceptance of emotional reactions, difficulties with engaging in goal-directed behavior, limited access to ER strategies, and difficulties with impulse control. The cognitive domains involved in this moderated relationship with stress were primarily attention^21,68,69^ language^68,70^, and memory^68,69,71^ (which have all been previously found to be associated with chronic stress). With further increase in age (over 65 years), the relationship between stress and cognition was no longer dependent on ER and was present across most cognitive domains. These findings indicate that men and women differ in their ability to manage stress so that its manifestations and consequences do not negatively affect other areas of their functioning, and that the effectiveness of this coping decreases with age in men.

At the behavioral level, we argue that this difference is related to a different preference for stress coping strategies and their age-specific effectiveness. Previous studies have shown that men generally prefer problem-focused coping, which is more cognitively dependent, while women tend to favor emotion-focused coping^46–51^. The effectiveness of problem-focused strategies decreases with age due to a decline in coping and cognitive resources such as increased physiological vulnerabilities^72,73^ and leads to prolonged exposure to stronger physiological and behavioral stress responses. This effect is further exacerbated when stressors cannot be avoided or resolved (as in the many of today’s stressors such as the Covid-19 pandemic or the war in Ukraine, etc.)^73,74^. This is supported by our findings, as the relationship between stress and cognition emerged first in men who had ER problems in areas that largely involve the executive component (such as goal-directed behavior, impulse control, etc.) and in later life stress was directly negatively related to cognition again only in men. Moreover, ER itself represents a resource-demanding process, affecting simultaneously or subsequently performed tasks (such as coping with stressors)^75,76^. This leads to an age-related reduced ability to both cope with stress and its negative emotional effects. Furthermore, the observed smaller repertoire of ER strategies in men reduces (similarly to limited palette of coping strategies) the individual's resources for effective management of the experienced stress. Finally, a reduced ability to accept (stress-related) emotions, another observed ER dimension moderating the stress-cognition relationship, limits the potential for coping with these emotions because, first, the strategies used may not be properly targeted and second (consequently), unaddressed negative emotions maintain the organism's stress setting for an extended period of time with all its negative behavioral and physiological consequences^77–80^.

From a wider interpretative perspective, neurophysiological findings from previous studies on shared neural pathways and brain regions also substantiate the observed age- and sex-specific links between ER, chronic stress, and cognition. For instance, the neural stress response was shown to be associated with a sex-specific pattern of activation of different brain regions. Men primarily activate cognitive control-related frontal areas, while women primarily activate the more emotion-associated limbic system^52,81–83^. Some studies have suggested that stress disrupts cognitive regulation of emotion linked to prefrontal cortex (PFC) activity, which limits the use of PFC in controlling the affective response to stress^84^. Poorer ability to control the emotional response to stress and an age-related decline in the utilization of the PFC in men then result in the reduced ability to mitigate the negative impact of stress on cognition. Furthermore, the prefrontal cortex, which is involved in problem-focused coping-associated executive functions, is particularly vulnerable to age-related decline^85–87^. In contrast, the amygdala and hippocampus, which are involved in emotional processing, are more resistant to age-related changes^88,89^. Finally, new theoretical models of ER propose that some of the ER processes during the stress response lead to an increase in inflammatory markers that may negatively affect other brain processes, including cognition^90–93^.

This study has several limitations. First, the data are based on self-reported instruments and the observed values are based on the subjective perception of the participants. Therefore, the obtained results do not, for example, provide information about underlying neurobiological processes and at the same time depend on the participants' level of awareness/self-reflection and their willingness to report any difficulties. For example, experimental measurement of emotion regulation in emotionally challenging situations could provide more accurate data with lower risk of bias due to differences in the ability to be aware of one's own emotional states. On the other hand, given the longitudinal nature of the Kardiovize study and the high engagement and motivation of participants, we consider that bias due to reluctance to report accurate and truthful information was negligible. Furthermore, regarding stress, it is worth noting that although there is a distinction between 'objective' stress (which manifests itself through the body's physiological responses and activation of neural pathways) and 'subjective' stress (involving emotional, cognitive, and behavioral responses to the stressor), the subjective perception of an event as overwhelming usually also activates the body's physiological stress response, especially in the context of chronic stress. Second, given their cross-sectional nature, all instruments measured how is currently experienced chronic stress related to immediate cognitive performance and did not allow causal inferences to be drawn. Third, although we controlled for potential selected psychopathologies (as exclusion criteria), and the Kardiovize sample was overall healthy, we cannot completely rule out the presence of other forms of psychopathologies that could affect the relationships studied. This potential influence should thus be kept in mind when interpreting the results. Fourth and finally, given the cross-sectional nature of the data, we did not assess possible bidirectional interactions between ER and cognitive performance (as cognitive performance may not only be the outcome in the observed relationship, but may also modulate the resources available to ER, creating a bidirectional loop). Again, future studies with longitudinal and/or experimental designs should examine this interplay in more detail.

The current study highlights several topics that ought to be explored further by future research. Given the cross-sectional nature of our results, longitudinal studies are needed to elucidate the long-term dynamics of the observed relationships. This includes both the impact of long-term stress on cognition and the role of ER in this interaction, and multidirectional interactions and feedback loops between the studied variables. Furthermore, these longitudinal studies could define the variation vs. stability of these relationships across different stratifications of time (e.g., changes over a few years in the context of different periods of adulthood), the rate of onset and extinction of direct stress and ER moderating effects, the point at which the direct stress effect overrides the moderating role of ER, etc. Finally, the data in this study were obtained using self-report measures. Given that emotion regulation can also be tested in experimental modes (e.g., directing participants to regulate emotions in emotionally challenging situations), and similarly stress can be probed at the level of physiological responses or activation of neural correlates, incorporating these measures into the methodology of future studies may confirm and extend these findings.

These results may have also practical implications for interventions. First, they emphasize the importance of considering age, sex, and individual differences in ER when considering strategies for prevention, intervention, care, and support. Second, they identify areas of emotion regulation that may represent a weak point with increasing age, particularly in men, in the context of coping with the impact of chronic stress on cognition. For example, targeted interventions and training in these ER skills, such as cognitive-behavioral therapy or mindfulness-based interventions, as part of chronic stress management care may improve coping with chronic stress and thereby reduce its negative consequences (even beyond cognition). Proactively, these efforts would not have to focus only on the adult population. For example, expanding school curricula to include activities that develop ER skills can have a significant positive developmental and preventive effect. Similarly, more attention should be paid to raising public awareness of the importance of and ways to develop emotional regulation skills.

In conclusion, our study is one of the first to uncover substantial sex- and age-related differences in how ER affects the relationship between chronic stress and cognitive performance. The results showed that chronic stress is associated with reduced cognitive performance only in men later in life and highlighted that better ER skills may delay the onset of this negative relationship.

## Methods

### Design and procedures

The sample included participants of the Kardiovize study, a longitudinal epidemiological cohort based on a representative randomly selected 1% population sample of the residents of Brno, Czech Republic, which investigates health-related topics in Central Europe^62,94^ This study initially included 582 participants who completed the second wave of data collection (conducted between 2021 and 2022), which involved measurement of all three variables: perceived stress, cognitive performance, and ER difficulties. Exclusion criteria involved incomplete data and the presence of specific psychopathologies (dementia, ongoing major depressive disorder, personality disorders, schizophrenia) that could substantially affect the relations studied. None of the participants showed the presence of psychopathology, 13 cases were excluded due to incomplete data, resulting in a final sample of 569 participants.

To investigate the proposed relationships at different stages of adulthood and to test these hypotheses, we stratified the samples into three age groups: 33–49 years, 50–64 years and 65 + years. This stratification was determined based on several developmental and transitional aspects that are critical to understanding how cognitive abilities may be influenced by both stress and emotion regulation. Individuals aged 33–49 years are experiencing peak cognitive abilities^95^, developing and refining their emotion regulation strategies and biological changes related to aging are relatively minimal during this period. Individuals in this age are often dealing with career development, family responsibilities, and personal growth and stressors are mainly related to the demands of work and family life. Between the ages of 50 and 64, cognitive changes become more apparent with shifts in memory, processing speed, and other cognitive functions may begin to manifest^96^, and emotional regulation skills are already well consolidated. Developmentally, this period is associated with a number of life changes such as children leaving home, potential career shifts, and the beginning of retirement planning, which changes the composition of stressors^97,98^. These may also include concerns about health and aging. After the age of 65, cognitive decline is more evident^61^. Retirement and related lifestyle changes, pension and financial security, biological aging, and health issues become major concerns and potential sources of stress. The ability to cope with these stressors (and their emotional accompaniments) also plays a key role^58,99^. In addition, we also sought to balance the sample sizes of each group as part of the statistical considerations.

### Measures

Stress was measured using 10-item Perceived Stress Scale (PSS-10)^100^. This instrument measures prolonged perceived stress (considered chronic) as the time frame of the items covers the last month. Scores range from 0 to 40, with higher values representing greater levels of perceived stress. Reliability of PSS was good with Cronbach’s alpha ranging from 0.74 to 0.91 ^101^.

Cognitive performance was measured using the MoCA screening tool^102^. We calculated six Cognitive Domain Index Scores (CDIS) to capture performances in attention (range 0–13, higher score always represents better performance), orientation (range 0–6), memory (range 0–15), language (range 0–6), visuospatial abilities (range 0–7) and executive functions (range 0–13)^103^. Cognitive performance in individual CDIS was measured using a series of varied items including trail-making, cube and clock drawing, animal naming, repetition of words, numbers and sentences, number subtraction, word matching, and semantic word similarity. The potential ceiling effect of using this screening tool in a healthy population was taken into account but was not considered to be a risk of biasing the results, as it allowed, on the contrary, to capture particularly the relationships between stress/ER and more pronounced cognitive decline. MoCA showed high test–retest reliability (ICC = 0.92, *P* < 0.001), and a good internal consistency (α = 0.83).

Emotion regulation was measured using Difficulties in Emotion Regulation Scale (DERS-18)^104^. This 18-item questionnaire measures the level of ER difficulties in six domains: non-acceptance of emotional responses (Non-acceptance), difficulty engaging in goal-directed behavior (Goals), impulse control difficulties (Impulse), lack of emotional awareness (Awareness), limited access to ER strategies (Strategy) and lack of emotional clarity (Clarity). The total scale ranges from 18 to 90, with higher (total and domain) scores representing greater difficulties with ER. DERS-18 proved to have excellent reliability with α = 0.91.

In addition, several socio-demographic characteristics were collected, including sex, marital status, education and household income.

### Statistical analyses

There were no missing values in the data. A Pearson correlation was used to perform an initial analysis of the relationship between perceived stress and cognitive performance. Differences in the presence of ER difficulties and in the levels of selected CDIS between age groups and between sexes were analyzed using two-way ANOVAs with partial eta-squared as effect size indicator (≥ 0.01 = small, ≥ 0.06 = moderate, ≥ 0.14 = large effect). The moderating role of ER difficulties in the influence of stress on cognitive performance was analyzed using multiple regression with interaction. The values of stress level (independent variable) and ER difficulties (moderator) were centered prior to the moderation analysis. Standardized betas with standard errors of simple main effects and interactions are reported in the text.

All statistical analyses were performed as a two-tailed and all the *P* < 0.05 were considered statistically significant. Data were analysed with RStudio (v.2022.07.2 with R environment v.4.2.1) using ggplot2, gridExtra, stats, interactions, and effectsize packages.

### Ethical statement

The authors assert that all procedures contributing to this work comply with the ethical standards of the relevant national and institutional committees on human experimentation and with the Helsinki Declaration of 1975, as revised in 2008. The research protocol was approved by St. Anne’s University Hospital ethics committee and the Internal Review Board. All participants were acquainted with the research and provided written informed consent.

### Supplementary Information

Supplementary Tables.

## Data Availability

The data used in this study are available on request immediately following the publication to anyone who submits the online request (mail: juan.gonzalez@fnusa.cz) including research intentions and goals that will be approved by the St. Anne’s University Hospital International Clinical Research Centre internal board.
